# Protective Effect of Lemon Essential Oil and Its Major Active Component, D-Limonene, on Intestinal Injury and Inflammation of *E. coli*-Challenged Mice

**DOI:** 10.3389/fnut.2022.843096

**Published:** 2022-06-02

**Authors:** Chen Zhao, Zhuo Zhang, Dechao Nie, Yanling Li

**Affiliations:** Animal Science and Technology College, Beijing University of Agriculture, Beijing, China

**Keywords:** lemon essential oil, d-limonene, intestinal injury, anti-inflammation, mice

## Abstract

Inflammatory diseases are a major threat to public health. Natural plant essential oils (EOs) possess anti-inflammatory and anti-oxidative activities. The objective of this study was to investigate the anti-inflammatory effect and mode of action of lemon essential oil (LEO), and its main active component, d-limonene, with different doses on intestinal inflammation of mice. Sixty-four 5-week-old male balb/c mice weighing 22.0 ± 1.5 g were randomly assigned into one of 8 treatments (*n* = 8/treatment), including normal saline group (NS), *Escherichia coli* (*E. coli*) group, and either LEO and d-limonene essential oil (DEO) group supplemented at 300, 600, and 1,200 mg/kg of BW, respectively. After the pre-feeding period, the mice were fasted for 12 h, the mice in the NS group and the *E. coli* group were gavaged with normal saline, and the mice in the LEO group and DEO group were gavaged with respective dose of EOs for 1 week. One hour after the end of gavage on the 7th day, except that the mice in the normal saline group were intraperitoneally injected with normal saline, the mice in the other groups were intraperitoneally injected with the same concentration of *E. coli* (10^8^ cfu/ml, 0.15 ml per mouse). The antioxidant indexes were measured including superoxide dismutase (SOD), malondialdehyde (MDA), and myeloperoxidase (MPO) in plasma obtained by taking blood from mouse eyeballs. The inflammatory indexes were measured including interleukin-6 (IL-6), interleukin-1β (IL-1β), and tumor necrosis factor alpha (TNF-α) in plasma. The tight junction protein indicators were tested include zona occludens 1 protein (ZO-1), occludin and claudin in mouse duodenum. We found that all of the above indexes for *E. coli* group were different (*P*< *0.05*) with the NS group. The interaction of EO and dose (E × D) were significant (*P* < 0.01) for all of the indexes. In addition, LEO at 300 mg/kg BW and DEO at 600 mg/kg BW had better antioxidant and anti-inflammation activity on the infected mice, which reduced (*P* < 0.05) the plasma concentrations of MDA, MPO, TNF-α, IL-1β, and IL-6, but increased (*P* < 0.01) the concentrations of SOD. Hematoxylin-eosin (H&E) staining of duodenum observation showed that LEO and DEO reduced inflammatory cell infiltration and maintain the orderly arrangement of epithelial cells. Moreover, supplementation of LEO at 600 mg/kg and DEO at 300 mg/kg BW alleviated (*P* < 0.05) intestinal barrier injury for increasing the relative expression of ZO-1, occludin and claudin mRNA in mice duodenum. These results showed that the pre-treatment with LEO and DEO had protection of intestinal tissue and inflammation in *E. coli* infected mice. Both LEO and DEO exhibited activity of antioxidant, anti-inflammatory and alleviating intestinal injury, whereas, compared with DEO, LEO can be active at a lower dosage. Furthermore, as the main active component of LEO, the d-limonene appeared to play not only the major role, but also the joint action with other active components of LEO.

## Introduction

Intestinal mucosa is a channel to absorb nutrients from metabolites of food and microorganisms, and also a barrier to prevent microorganisms from invading tissues and alleviate the inflammatory response to a large amount of contents in the cavity (1). When the intestinal epithelial barrier is damaged, the intestinal permeability increases, and harmful substances enter the blood stream, which induces inflammatory response and endangers the health of the host (2). Accordingly, when animals encounter the harm of pathogenic microbial infection and oxidative stress, which reduces their production performance, and even lead to death. At present, antibiotics are widely used to promote intestinal health, but the toxicity and side effects limit their application (3, 4), and many countries prohibit the use of antibiotics as growth promoting substances. It is a hot topic to enhance the intestinal barrier function and improve the health status of animals by means of nutritional regulation. Therefore, it is very important to look for natural, safe and efficient feed additives that can replace antibiotics in the field of animal nutrition.

Essential oils (EOs) could promote the growth of intestinal probiotics and inhibit the proliferation of pathogenic bacteria, so as to improve the structure of intestinal flora (5). Citrus genus, the most important genus in the Rutaceae family, is found in many countries, besides Brazil, Japan, Argentina, USA, and Australia (6). Lemon essential oil (LEO), as one of the citrus EOs, extracted from lemon fruit has been used to treat a variety of pathological diseases, such as inflammation, cardiovascular disease, cancer, hepatobiliary dysfunction and so on (7, 8). LEO has previously been reported to possess multiple biological activities and is considered as generally recognized as safe (GRAS) by FDA 2018 (9). It was showed that LEO had antibacterial and fungicidal properties on foodborne pathogens and spoilage bacteria (10, 11), and also considerable antioxidant properties (12). Studies have shown that citrus EOs exhibited a selective antibacterial activity with higher effect on pathogenic bacteria (*Escherichia coli, E. coli*) than beneficial bacteria (*Lactobacillus*) (13). All of this suggests the potential of LEO to be used as a natural extract for preventing inflammation caused by oxidative stress. However, to the best of our knowledge, few studies have reported the action mechanisms of LEO on the inflammatory mice.

The d-limonene was showed as the main active component of LEO (14). Here, we compared the effects of LEO and DEO to further explore whether LEO relied on its main active component d-limonene to exert its anti-inflammatory activity. This study aimed to provide a theoretical basis for the rational application of LEO in animal production by exploring the effects of different concentrations of LEO on intestinal barrier and inflammatory response in *E. coli* injected mice.

## Results

### Component Analysis of EOs

The active component profiles of testing LEO and DEO in this experiment are shown in Tables 1, 2, respectively. There were 30 active components being detected in LEO, of which the proportion of d-limonene was the highest (47.5% of total), and followed by β-pinene (14.69% of total), γ-terpinene (9.61% of total), β-laurene (4.89% of total) and α-pinene (4.28% of total). Total 20 active components were detected in DEO, of which d-limonene was the primary active component (83.5% of total).

**Table 1:** Main chemical components and relative contents of LEO.

**Peak**	**RI/min**	**Name**	**Area%**
1	6.919	Bicyclo[3.1.0]hex-2-ene, 2-methyl-5-(1-methylethyl)	0.59
2	7.127	Alpha.-Pinene	4.28
3	7.516	Camphene	0.26
4	8.396	β-pinene	14.69
5	8.665	Beta.-Myrcene	4.89
6	9.036	1,6-Octadien-3-ol, 3,7-dimethyl-, formate	1.14
7	9.314	(+)-4-Carene	0.85
8	10.057	D-Limonene	47.48
9	10.586	Gamma.-Terpinene	9.61
10	10.815	Cyclohexene, 4-methyl-1-(1-methylethenyl)	0.21
11	11.159	Cyclohexene, 1-methyl-1-(1-methylethenyl)	1.27
12	11.535	Linalool	0.38
13	13.908	Alpha.-Terpineol	0.39
14	14.175	Decanal	0.29
15	15.000	Beta.-Citral	3.55
16	15.719	Alpha.-Citral	4.03
17	17.718	Nerol acetate	0.99
18	18.150	Geranyl acetate	0.87
19	18.409	Cyclohexane, 1-ethenyl-1-methyl-2,4-bi	0.13
20	19.059	Caryophyllene	0.40
21	19.268	Gamma.-Elemene	0.67
22	19.825	Humulene	0.20
23	20.414	Bicyclo[7.2.0]undec-4-ene, 4,11,11-trim	0.15
24	20.600	Naphthalene, 1,2,3,5,6,7,8,8a-octahydro-1,8a-dimethyl-7-(1-methylethenyl)-, [1R-(1.alpha.,7.beta.,8a.alpha.)]	0.21
25	20.840	Alpha.-Farnesene	1.16
26	23.779	Triethyl citrate	0.56
27	28.548	Cyclohexane, 1-methylene-4-(1-methyle)	0.13
28	28.944	M-Camphorene	0.32
29	29.523	P-Camphorene	0.20
30	31.373	(S,E)-2,5-Dimethyl-4-vinylhexa-2,5-die	0.12
Total			100

**Table 2:** Main chemical components and relative contents of DEO.

**Peak**	**RI/min**	**Name**	**Area%**
1	7.096	Alpha.-Pinene	2.87
2	8.138	Bicyclo[3.1.0]hexane, 4-methylene-1-(1-methylethyl)	2.07
3	8.627	Beta.-Myrcene	2.70
4	8.673	Beta.-Myrcene	4.11
5	9.015	Octanal	0.29
6	9.078	Beta.-Ocimene	1.36
7	10.149	D-Limonene	83.52
8	10.252	Cis-.beta.-Ocimene	0.10
9	10.495	Gamma.-Terpinene	0.38
10	11.143	Cyclohexene, 3-methyl-6-(1-methylethy)	0.15
11	11.529	Linalool	0.55
12	12.077	1R,4R-p-Mentha-2,8-dien-1-ol	0.14
13	12.446	Cis-p-Mentha-2,8-dien-1-ol	0.12
14	13.494	2-Isopropenyl-5-methylhex-4-enal	0.09
15	13.968	Cis-Dihydrocarvone	0.13
16	14.483	Alpha.,4-Dimethyl-3-cyclohexene-1-acetaldehyde	0.20
17	14.899	P-Menthane, 1,2:8,9-diepoxy	0.10
18	15.068	(-)-Carvone	0.52
19	16.082	Anethole	0.10
20	43.279	Tris(2,4-di-tert-butylphenyl) phosphate	0.52
Total			100

### LD_50_ of EOs

According to the modified Karber's method (15), the LD_50_ of mice with oral administration of LEO was observed to be 3,162 mg/kg of BW, with 95% confidence interval of 2,934.27–3,408.00 mg/kg of BW (Table 3). The LD_50_ of mice with oral administration of DEO was 3,198.89 mg/kg, with 95% confidence interval of 2,904.69–3,522.89 mg/kg.

**Table 3:** Acute oral toxicity of essential oils in mice.

**EOs**	**Group**	**Dose (mg/kg)**	**Animals (numbers)**	**Mortality**	**Survival rate**
LEO	1	1500.00	10	0.00	1.00
	2	2040.00	10	0.10	0.90
	3	2774.40	10	0.30	0.70
	4	3773.20	10	0.60	0.40
	5	5131.50	10	1.00	0.00
DEO	1	1,600.00	10	0.00	1.00
	2	2240.00	10	0.20	0.80
	3	3136.00	10	0.40	0.60
	4	4390.00	10	0.80	0.20
	5	6146.00	10	1.00	0.00

*LEO, lemon essential oil; DEO, d-limonene essential oil.*

### Effect of EOs on Plasma Antioxidant Indexes of *E. coli* Infected Mice

**Figure 1:**
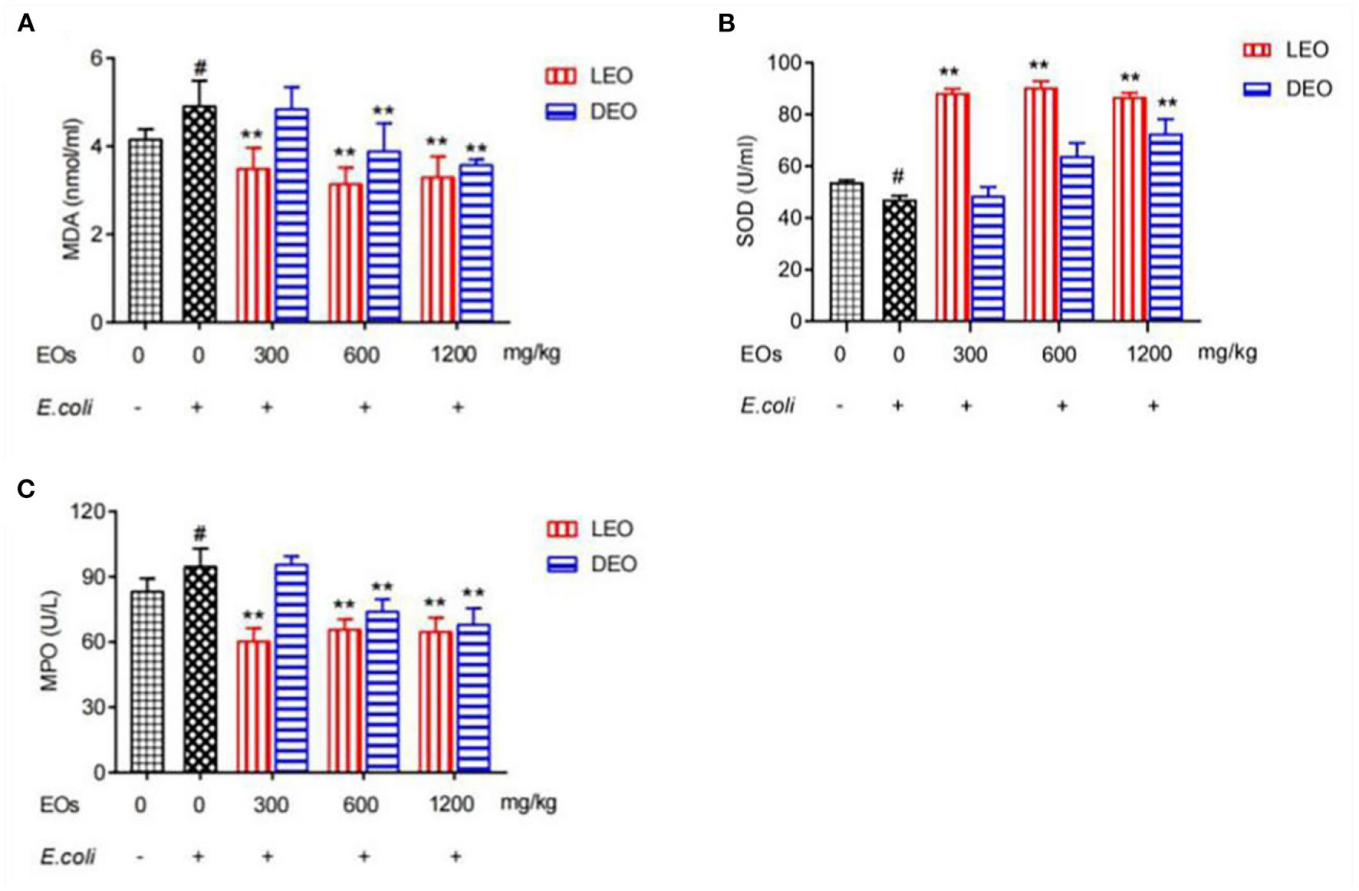
Effects of LEOand DEO on plasma *concentration* of malondialdehyde [MDA; **(A)**], superoxide dismutase [SOD; **(B)**], and myeloperoxidase [MPO; **(C)**] in *E. coli* injected mice. Data were presented as mean ± standard error of mean (SEM) (*n* = 8). The 1st column was normal saline group (NS) with no *E. coli* and no EO. The 2nd column was the *E. coli* group with no EO. The 3rd−8th column are the LEO and DEO groups with doses at 300, 600, and 1,200 mg/kg BW, respectively. #*P* < 0.05 *E. coli* group compared with NS group. Essential oil (E) and its different doses (D) had significant effects on plasma antioxidant indexes (*P*_*E*_ < 0.01, *P*_*D*_ < 0.01) for MDA, SOD, and MPO, and their interaction (E × D) was significant (*P*_*E*_ × *P*_*D*_ < 0.01). **P < 0.01, EO groups compared with *E. coli* group. LEO, lemon essential oil; DEO, d-limonene essential oil.

Effects of EOs on plasma antioxidant indexes of *E. coli* infected mice were showed in Figure 1. Compared with normal saline (NS) group, *E. coli* group increased (*P* < 0.05) plasma concentrations of malondialdehyde (MDA) and myeloperoxidase (MPO), but decreased (*P* < 0.05) concentration of superoxide dismutase (SOD). Supplementation of LEO and DEO had significant influence (*P* < 0.01) on the concentration of MDA, MPO, and SOD. The interaction of EO and dose (E × D) were significant (*P* < 0.01) for all of the three antioxidant indexes. Supplementation of LEO quadratically decreased (*P*_*Q*_ < 0.01) the concentration of MDA and MPO with increase dose (Figures 1A,C) and quadratically increased (*P*_*Q*_ < 0.01) the concentration of SOD with increase dose (Figure 1B). Supplementation of DEO linearly decreased (*P*_*L*_ < 0.01) the concentration of MDA and MPA with increase dose (Figures 1A,C) and linearly increased (*P*_*L*_ < 0.01) the concentration of SOD with increase dose (Figure 1B). The concentration of MDA for LEO group was lower (*P* < 0.01) than that for DEO group at 300 mg/kg. The concentrations of MPO for LEO group were lower (*P* < 0.01; *P* = 0.02) than those for DEO group at 300 and 600 mg/kg, respectively. The concentrations of SOD for LEO group was higher (*P* < 0.01) than that for DEO group at 300 and 600 mg/kg, respectively.

### Effect of EOs on Duodenum Morphology of *E. coli* Infected Mice

**Figure 2:**
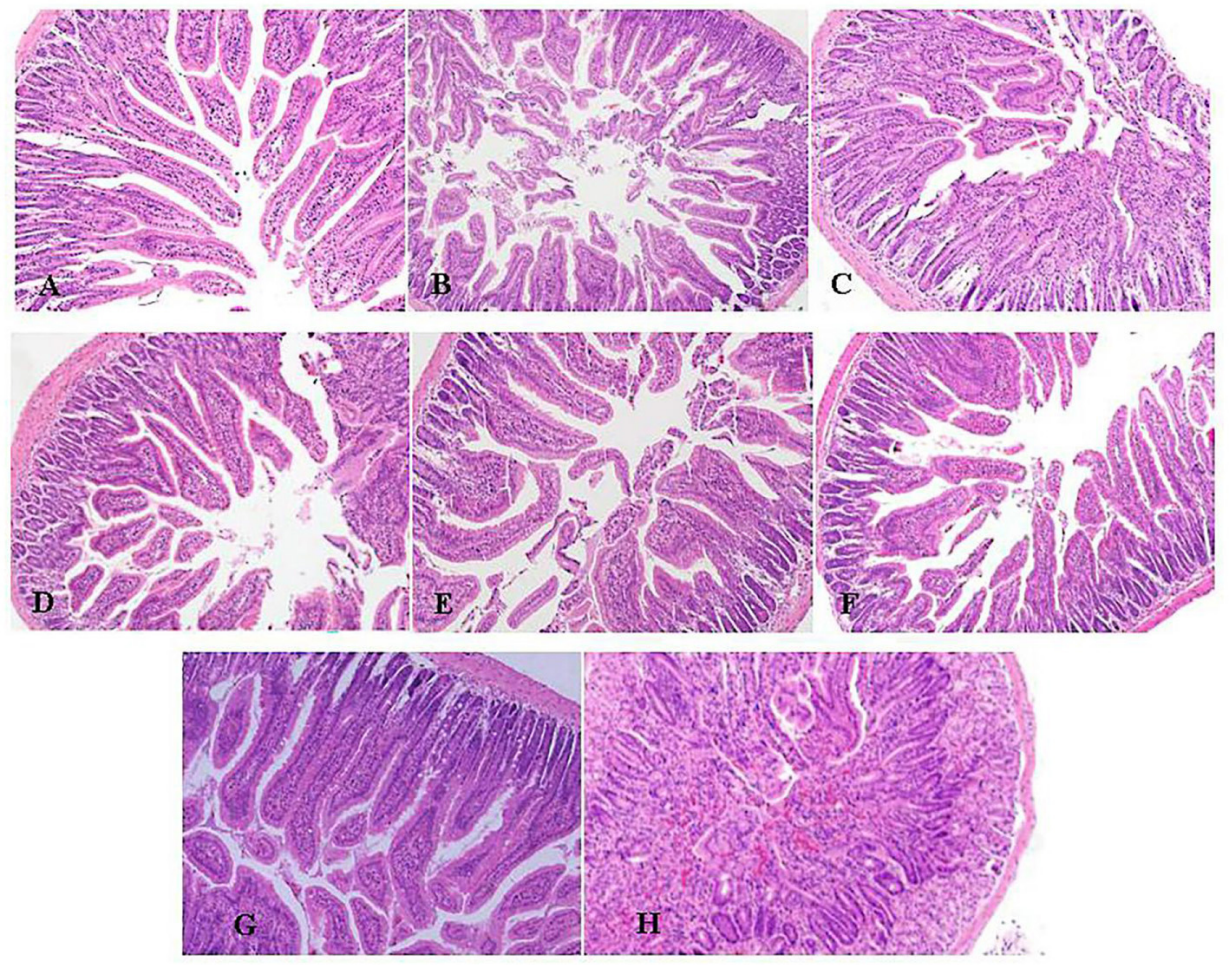
Effects of varying doses of LEO **(A–C)** or DEO **(D–F)** from 300, 600 to 1,200 mg/kg BW on pathological of H&E stained duodenum (100 ×) in *E. coli*-infected mice **(A–C)**. **(G)**: NS group. **(H)**: *E. coli* group. LEO, lemon essential oil; DEO, d-limonene essential oil.

After *E. coli* infection, the duodenal structure of mice was damaged, including increased nuclear aggregation, increased inflammatory cells, disordered glands and sparse villi showed as Figure 2H compared with the NS group showed as Figure 2G. The pathological changes were improved and the inflammatory cell infiltration was reduced with pretreatment of LEO (Figures 2A–C) and DEO (Figures 2D–F). The pretreatment with LEO and DEO improved the intestinal barrier injury even at the low dose.

### Effect of EOs on Plasma Inflammatory Factors of *E. coli* Infected Mice

**Figure 3:**
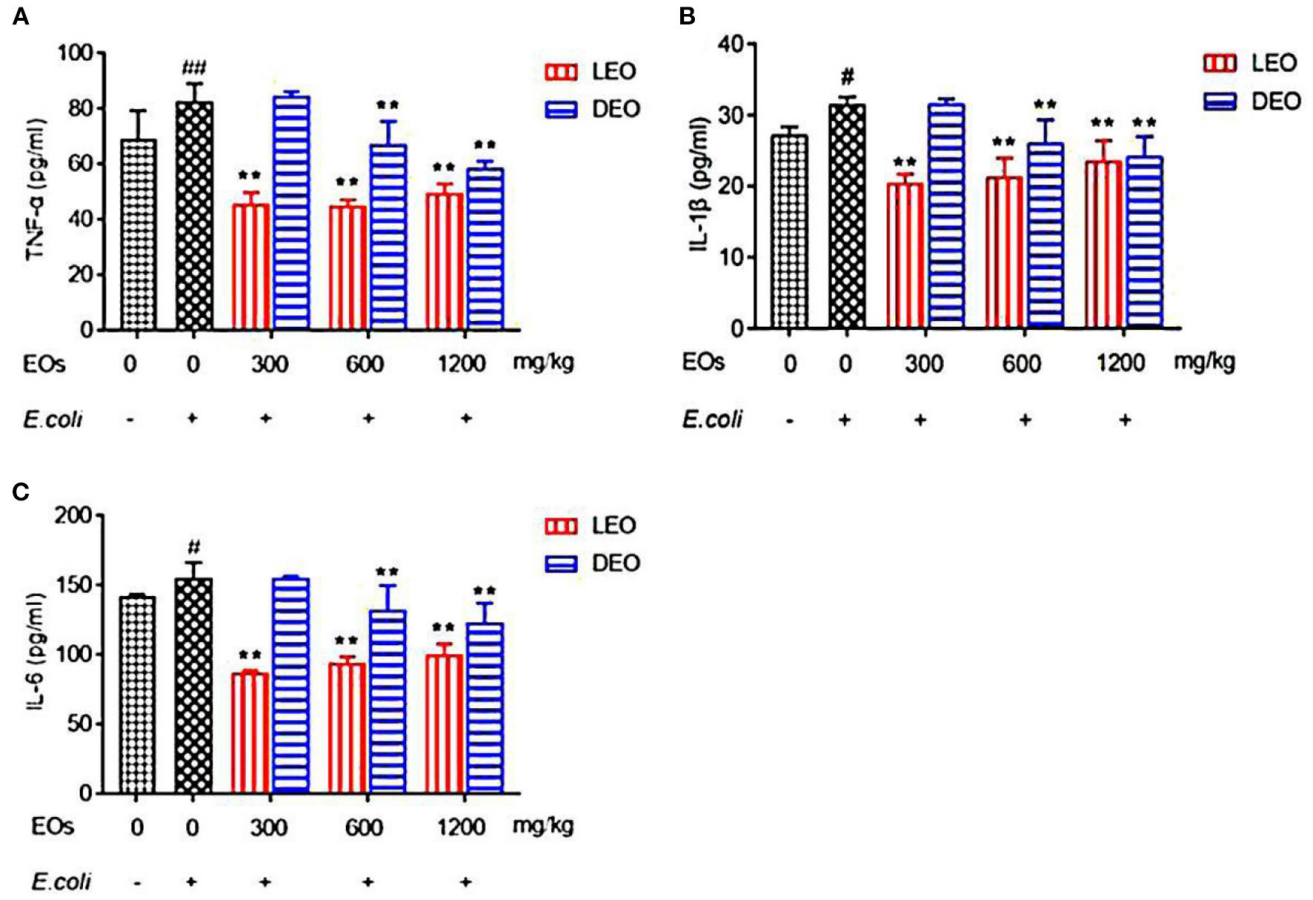
Effects of LEO and DEO on plasma concentration of tumor necrosis factor α [TNF-α; **(A)**], interleukin-1β [IL-1β; **(B)**], and interleukin 6 [IL-6; **(C)**] in *E. coli* injected mice. Data were presented as mean ± standard error of mean (mean ± SEM) (*n* = 8). The 1st column was normal saline group (NS) with no *E. coli* and no EO. The 2nd column was the *E. coli* group with no EO. The 3rd−8th column are the LEO and DEO groups with doses at 300, 600, and 1,200 mg/kg BW, respectively. #*P* < 0.05 and ##*P* < 0.01, *E. coli* group compared with NS group; Essential oil (E) and its different dose (D) had significant effects on plasma inflammatory factors (*P*_*E*_ < 0.01, *P*_*D*_ < 0.01), and their interaction (E × D) was significant (*P*_*E*_ × *P*_*D*_ < 0.01). **P < 0.01, EO groups compared with *E. coli* group. LEO, lemon essential oil; DEO, d-limonene essential oil.

Effects of EOs on plasma inflammatory factors of *E. coli* infected mice were showed in Figure 3. Compared with NS group, *E. coli* group increased (*P* < 0.05) the plasma concentrations of tumor necrosis factor alpha (TNF-α), interleukin-1β (IL-1β) and interleukin-6 (IL-6) (Figure 3). Supplementation both of LEO and DEO decreased (*P* < 0.01) the concentration of TNF-α, IL-1β, and IL-6. The interaction of EO and dose (E × D) were significant (*P* < 0.01) for all of the three inflammatory factors. Supplementation of LEO and DEO quadratically decreased (*P*_*Q*_ < 0.01) the concentration of TNF-α, IL-1β, and IL-6 with increase dose, respectively (Figures 3A–C). The concentrations of TNF-α, IL-1β, and IL-6 for LEO group were lower (*P* < 0.01) than those for DEO group at 300, 600, and 1,200 mg/kg, respectively.

### Effect of EOs on the Relative Expression of Duodenal Tight Junction Protein mRNA in *E. coli* Infected Mice

**Figure 4:**
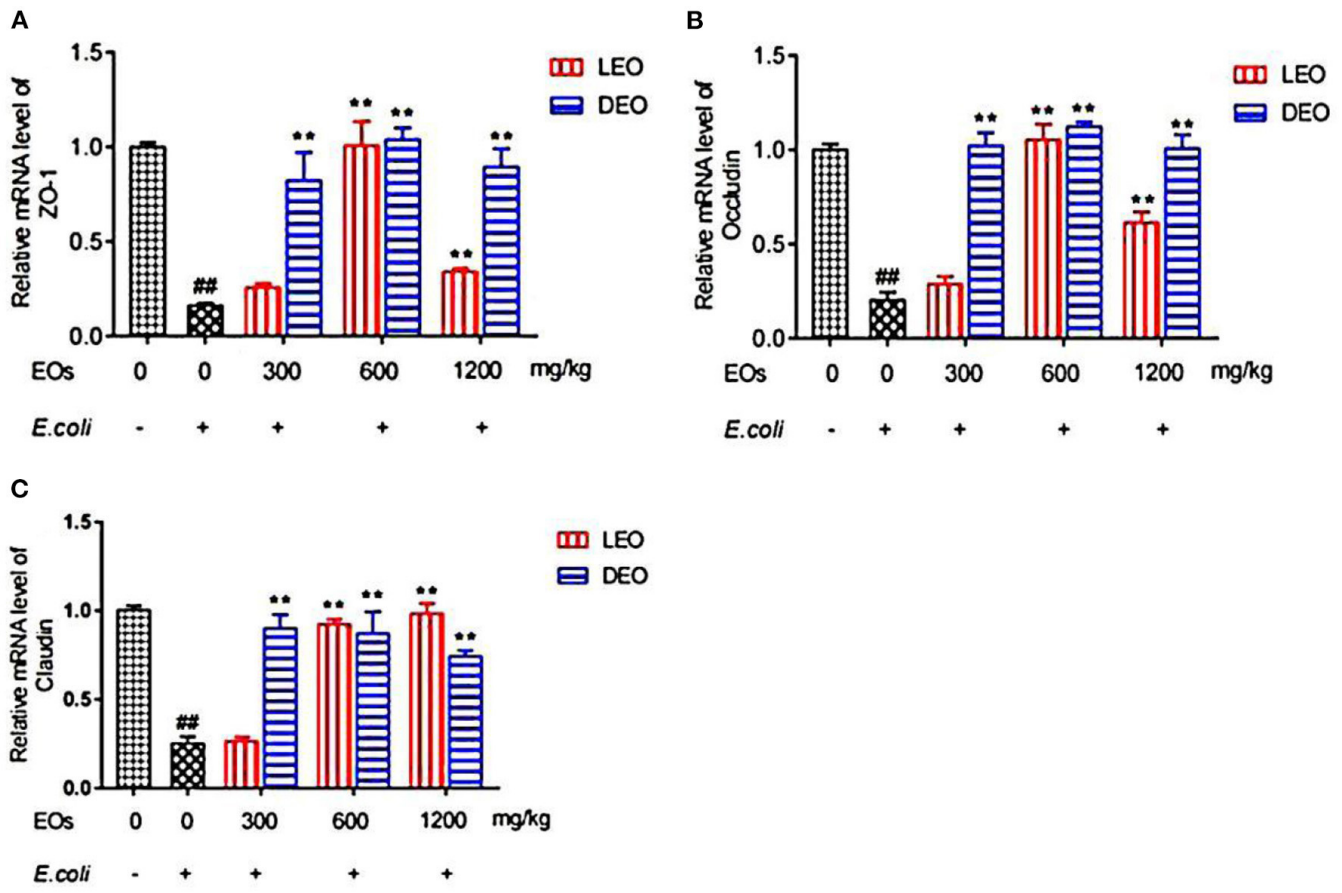
Effects of LEO and DEO on secretion of ZO-1 **(A)**, occludin **(B)** and claudin **(C)** in *E. coli* injected mice duodenum. Data were presented as mean ± standard error of mean (SEM; *n* = 8). The 1st column was normal saline group (NS) with no *E. coli* and no EO. The 2nd column was the *E. coli* group with no EO. The 3rd−8th column are the LEO and DEO groups with doses at 300, 600, and 1,200 mg/kg BW, respectively. ##*P* < 0.01, *E. coli* group compared with NS group; Essential oil (E) and its different dose (D) had significant effects on duodenal tight junction proteins (*P*_*E*_ < 0.01, *P*_*D*_ < 0.01), and their interaction (E × D) was significant (*P*_*E*_ × *P*_*D*_ < 0.01). **P < 0.01, EO groups compared with *E. coli* group. LEO, lemon essential oil; DEO, d-limonene essential oil.

Effects of EOs on the relative expression of duodenal tight junction protein mRNA of *E. coli* infected mice were showed in Figure 4. Compared with NS group, *E. coli* group reduced (*P* < 0.01) the relative mRNA expression of ZO-1, occludin and claudin (Figures 4A–C). Supplementation both of LEO and DEO increased (*P* < 0.01) the relative expression of ZO-1, occludin and claudin mRNA in mice (Figures 4A–C). The interaction of EO and dose (E × D) were significant (*P* < 0.01) for all of the three tight junction proteins. Supplementation of LEO quadratically increased (*P*_*Q*_ < 0.01) the concentration of ZO-1, and occludin with increase dose (Figures 4A,B) and linearly increased (*P*_*L*_ < 0.05) the concentration of claudin with increase dose (Figure 4C). Supplementation of DEO quadratically increased (*P*_*Q*_ < 0.05; *P*_*Q*_ = 0.02; *P*_*Q*_ = 0.02) the concentration of ZO-1, occludin and claudin with increase dose, respectively. The concentrations of ZO-1, and occludin for LEO group were lower (*P* < 0.01; *P* < 0.01) than those for DEO group at 300, 600, and 1,200 mg/kg, respectively. The concentration of claudin for LEO group was lower (*P* < 0.05) than that for DEO group at 300 mg/kg, while the concentration of claudin for LEO group was higher (*P* < 0.05) than that for DEO group at 600 and 1,200 mg/kg, respectively.

## Discussion

The main mechanisms of intestinal injury include inflammatory response, oxidative stress and apoptosis. Oxidative injury accompanied by inflammatory injury occurred in *E. coli* infected intestinal mucosal injury. In this study, three indexes (SOD, MDA and MPO) in plasma were determined to judge the antioxidant activity of EOs on *E. coli* infected mice. Our results showed that the increased concentrations of MDA and MPO, and decreased activity of SOD in *E. coli* injected intestinal mucosal injury are consistent with previous finding (16). With the increase of additive dose, LEO showed its antioxidant activity in the form of quadratic curve and DEO in the form of linear enhancement. Obviously, our results showed that the overall antioxidant activities of LEO were higher than those of DEO at different doses. It was found that the mixture of LEO and a ginger extract substantially decreased the lipid peroxidation intensity in the liver and the brains of mice and increased the resistance of liver and brain lipids to oxidation and the activity of antioxidant enzymes in the liver (17). Abdel-Daim et al. (18) found that treatment with limonia oil at 100 and 200 mg/kg BW improved all the biochemical alterations in a dose-dependent manner, which could increase the concentration of SOD, and decrease the concentration of MDA. CampêLo et al. (19) found that LEO reduced the level of lipid peroxidation and increased the level of GSH and SOD, catalase and glutathione peroxidase in mouse hippocampus. Rashidian et al. (20) found that adding 200 and 400 μl/kg Basil EO reduced the colitis index and the level of MPO in rats. The Basil EO was considered to have a protective effect on colitis in rats induced by acetic acid. These previous findings demonstrated that the pretreatment of various EOs had obvious antioxidant activity and the potential to prevent the damage caused by oxidative stress. It was found that the antioxidant properties of individual EOs from Citrus limoniaL largely depend on their active components such as limonene, pinene, terpinene (21). In this study, the main component of LEO and DEO was d-limonene and the proportion of d-limonene was greatly higher with DEO than that with LEO, whereas the antioxidant activity seemed to be lower with DEO than that with LEO. In addition, LEO had a variety of other active components besides d-limonene. Therefore, we speculated that the antioxidant activity of LEO was not only from its main component d-limonene, but from the combined effect of other active components. The specific mechanism needs to be further explored.

Inflammatory response is the key cause of intestinal barrier function injury. Zhang et al. (22) showed that ectopic intestinal flora in mice could stimulate intestinal mucosal inflammation, lead to certain disorder for the intestinal barrier function and mucosal immunity, and thus cause immunosuppression. The release of proinflammatory cytokines may lead to the interruption of tight junction and further damage the intestinal barrier (23). Therefore, reducing the release of inflammatory factors plays an important role on alleviating intestinal mucosal injury. By observing the effect of nutmeg EO on human fibroblasts, Matulyte et al. (24) reported that nutmeg EO had anti-inflammatory effect, protected cell viability and reduced the release of IL-6. Limonene in citrus EO inhibited TNF-α, IL-1β, IL-8, and IL-10 to inhibit p38 mitogen activated protein kinase signaling pathway and regulate chemokine induced chemotaxis (25). In addition, limonene might reduce the infiltration of monocytes and eosinophils. Amorim et al. (21) found that pretreatment with LEO reduced IL-1β and TNF-α in mice stimulated by carrageenan at the dose of 30 or 100 mg/kg, and decreased the production of IFN-γ at a high dose. Maurya et al. (26) showed that citrus peel EO inhibited the expression of pro-inflammatory cytokines of TNF-α, IL-6, and IL-1β in LPS-induced macrophage. Those authors speculated that the anti-inflammatory activity of citrus peel EO may be the result of limonene component (91.8%). Furthermore, Wattenberg and Coccia (27) reported that d-limonene and citrus EOs could inhibit NNK-induced carcinogenesis when they were administered 1 h prior to carcinogen challenge, suggesting that the inhibitory effects of these two EOs might be accounted for the presence of d-limonene. The present results in our experiment showed the reduction in the concentration of proinflammatory factors by LEO at the dose of 300 mg/kg, and by DEO at 600 mg/kg. Our results indicated that lower dose of LEO could inhibit inflammatory factors, but higher dose was needed for DEO, which suggest that the anti-inflammatory activity of LEO was not solely due to its main active component D-limonene. According to the GC-MS results, we found that the components of LEO include β-pinene, γ-terpinene, β-laurene, and α-pinene (33.47% of total), in addition to d-limonene. Therefore, we speculated that the better role of LEO in inhibiting inflammatory factors was due to the interaction of its complex active components.

Intestinal mechanical barrier, also known as physical barrier, is an intestinal epithelial structure composed of intestinal mucosal epithelial cells and their tight connections (28). It is an important barrier to prevent harmful substances such as virulence factors of intestinal pathogens from entering the blood circulation and even tissues and organs. The function of intestinal barrier can usually be evaluated by many indicators, such as serum endotoxin level, intestinal morphology and intestinal tight junction protein (29). Acheampong et al. (30) found that EO from citrus aurantifolia could improve histomorphological characteristics of testis, kidney, and liver of rats. Wang et al. (31) showed that atractylodes macrocephala EO significantly inhibited body weights loss, diarrhea, reductions of thymus and spleen indexes, and pathological changes of ileums and colons induced by 5-fluorouracil. In this study, mice infected with *E. coli* showed that typical pathological changes, including increased inflammatory cells, disordered arrangement and sparse villi, indicating that the duodenal structure was damaged. Our results showed that LEO and DEO had beneficial effects on reducing the infiltration of inflammatory cells, maintain the integrity of intestinal tract and protect animal body from damage. In addition, occludin, claudin and ZO-1 are the main proteins that play an important role in the tight junction of intestinal epithelial cells and the intestinal barrier (32). The tight junctions, represented by claudins, occludin, connective adhesion molecules and scaffold proteins, is mainly responsible for regulating paracellular transport and is also the main structural component forming the structural barrier function of epithelial cells (33, 34). They act as barriers and fences under normal and pathological conditions, preventing countless ions and small solutes from freely passing through the space between two interacting cells (35). Wang et al. (36) found that orange EO could regulate the intestinal microbiota of mice by increasing the relative abundance of Lactobacillus and reducing the presence of bacteria producing short chain fatty acids. EOs with the active ingredient of 13.5% thymol and 4.5% cinnamaldehyde markedly up-regulated the expression levels of occludin in the duodenum, which suggested that EOs maintained the integrity of intestinal barrier (37). Zou et al. (38) found that pretreatment with oregano EO could significantly decrease the expression of occludin and ZO-1 in porcine epithelial cells. Hui et al. (39) found that eugenol significantly inhibited the level of IL-8 and TNF-α stimulated by LPS and restore the mRNA abundance of tight junction proteins such as ZO-1 and occludin, so as to reduce inflammatory response and enhance the function of selective permeability barrier. Liu et al. (40) showed that oral administration of carvacrol EO could increase the gene expression of the occludin, claudin-1, claudin-5, ZO-1, and ZO-2 in intestinal mucosa and reduced the microbial counts of Salmonella spp. and *E. coli* in the intestines. In this study, both LEO and DEO could improve the expression of three tight junction proteins compared with *E. coli* group. Pretreatment with LEO and DEO increased relative expression of ZO-1, occludin and claudin, which demonstrated their potential role of improving tight junction and maintaining the integrity of intestinal barrier.

In conclusion, the citrus EOs exhibited anti-inflammatory, antioxidant activities and alleviated intestinal barrier damage. Both of LEO and DEO had positive effects on intestinal barriers function of mice. The pretreatment with LEO or DEO could protect the inflammation of *E. coli* infected mice by reducing the production of proinflammatory factors, increasing the concentration of antioxidant indexes and maintaining the integrity of duodenum. The anti-inflammatory effect of LEO appeared to be better than that of DEO whose main component was d-limonene when the same dose was applied. It speculated that besides of d-limonene, other active components in LEO may play significant role. Our work will shed light on developing new additive with anti-inflammatory activity from LEO.

## Materials and Methods

### Chemicals and Reagents

*Escherichia coli* (ATCC 25922) was purchased from China General Microbiological Culture Collection Center (Beijing, China). TNF-α, IL-6, and IL-1β enzyme-linked immunosorbent assay (ELISA) kit were provided by Beijing SINO-UK Institute of Biological Technology (Beijing, China). All other reagents were of analytical grade.

### Essential Oils and Active Component Analysis

Two EOs lemon oil (purity of 88%) and D-limonene (purity, 90%), with density 0.8468 and 08414 g/ml, respectively, were used in the present study [Nanjing Vincero International Trade Co., Ltd (Nanjing, China)].

The EO samples were diluted with n-hexane before analysis and their active components were determined using a GC-MS QP2010 ultra (Shimadzu, Kyoto, Japan). The active compounds were separated on a Rxi-5Sil MS (30 m × 0.32 mm inner diameter, 0.25 μm film thickness, Restek, USA) capillary column. The column temperature was initially set at 50°C for 5 min, then increased at a rate of 2°C /min up to 320°C, and held for 5 min. The injection volume was 1 μL, with a split ratio of 1:10. Helium was used as the carrier gas, at a flow rate of 1 mL/min. The injector, transfer line and ion-source temperatures were 250, 280, and 220°C, respectively. The MS detection was performed with electron ionization (EI) at 70 eV, operating in the full-scan acquisition mode in the m/z range 33–700.

### Animals

One hundred sixty-four balb/c mice (5-week old, weighing 22.0 ± 1.5 g) were purchased from the SPF Biotechnology Co., Ltd. (Beijing, China). Animals were housed at the temperature of 24°C and humidity ranging from (40 to 80%, with feed and water provided *ad libitum*. All experimental procedures were approved by the Laboratory Animal Ethics Committee of Beijing University of Agriculture, and conformed to the legal mandates and national guidelines for the care and maintenance of laboratory animals [SYXK (京) 2021-0001, 2021.01.04].

### Acute Toxicity Test of EOs

One hundred mice (50 male and 50 female) were randomly assigned into one of 10 groups (10 mice in each group with equal number of male and female). Five groups of mice were given LEO at dose of 1,500, 2,040, 2,774.4, 3,773.2, and 5,131.5 mg/kg BW once, respectively; second five groups were dosed with DEO at 1,600, 2,240, 3,136, 4,390, and 6,146 mg/kg BW/d, respectively by gavage. The survival rate of mice for 2 weeks was recorded. LD50 of EOs was calculated by modified Karber's method (15).

### Experimental Designs

Sixty-four mice were randomly assigned into eight treatment groups (*n* = 8 per group) as follows: (1) NS group (normal saline); (2) *E. coli* group (*E. coli*); (3–5) LEO group with supplementation rate at 300, 600, and 1,200 mg/kg BW, respectively; and (6–8) DEO group with supplementation rate at 300, 600, and 1,200 mg/kg BW, respectively. All mice were given EOs by oral gavage for 1 week, except those in the NS group and *E. coli* group that were given normal saline by oral gavage. After 1 h of the last administration gavage, all mice were injected intraperitoneally with *E. coli*, except those in the NS group that were injected intraperitoneally with normal saline. Mice were killed by cervical dislocation, and blood and duodenal samples were collected at 6 h after *E. coli* administration. The plasma collected from mouse eyeballs in anticoagulant tubes was used for determination of antioxidant indexes and inflammatory factors. The anterior segment of duodenum was collected for hematoxylin eosin (H&E) staining, and the posterior segment of duodenum was used for determination of the relative expression of tight junction protein mRNA.

### Determination of Plasma Antioxidant Indexes and Inflammatory Factors in Mice

The plasma antioxidant indexes and inflammatory factors were determined with enzyme-linked immunosorbent assay (41). Blood antioxidant indexes were characterized by analyzing antioxidant enzyme activities including SOD, MDA and MPO in plasma as described by using ELISA Kit (Beijing Sino-UK Institute of Biological Technology, Beijing, China). Inflammatory factors including TNF-α, IL-1β, and IL-6 were determined to assess the anti-inflammatory activities in plasma as described by using ELISA Kit (Beijing Sino-UK Institute of Biological Technology, Beijing, China).

### Histopathology

The samples of duodenum were fixed in 4% paraformaldehyde overnight at 4°C, embedded in paraffin and sectioned at 4-μm thickness (42). Tissue sections were stained with hematoxylin and eosin (H&E). The images were viewed and acquired using a microscope (ECLIPSE Ni-E, Nikon, Tokyo, Japan).

### RT-PCR

The duodenum obtained by mouse autopsy was stored at −70°C. The RNA of duodenum was extracted by RNA prep pure animal tissue total RNA Extraction Kit (DP431, TIANGEN). The RNA concentration, purity and integrity were measured at 260 and 280 nm on an ultra micro ultraviolet spectrophotometer, and then cDNA was prepared using GoScript™ Reverse Transcription System reverse transcription kit. Primer sequences were designed with software Primer 3.0 and synthesized by Sangon Biotech (Shanghai) Co., Ltd. (Table 4). Finally refer to TransStart® Top Green qPCR SuperMix was detected by real-time fluorescence quantitative PCR (RT-PCR). RT-PCR was performed with the reaction conditions as follows: pre-denaturalization at 95°C for 10 min. 40 cycles of 95°C 10 s, and 60°C 30 s. The β-actin was used as an internal reference for relative quantitative determination, and the results were expressed in the form of 2^−ΔΔCt^. The primers of ZO-1, occludin, claudin and β-actin are shown in Table 4.

**Table 4:** Primer sequences used in RT-PCR.

**Target**	**Primer sequence (5'-3')**
β-actin	F:GTGCTATGTTGCTCTAGACTTCG
	R:ATGCCACAGGATTCCATACC
ZO-1	F:GCGAACAGAAGGAGCGAGAAGAG
	R:GCTTTGCGGGCTGACTGGAG
Occludin	F:TGGCTATGGAGGCGGCTATGG
	R:AAGGAAGCGATGAAGCAGAAGGC
Claudin	F:GCTGGGTTTCATCCTGGCTTCTC
	R:CCTGAGCGGTCACGATGTTGTC

### Statistical Analysis

Data on the effect of *E. coli* stimulated mice, were analyzed using the general linear model (GLM) procedure of SAS 9.4 (SAS Inst. Inc., Cary, NC) to compare the *E.coli* group with the NS group. Statistical significance was assessed in pairwise comparisons using one-way ANOVA. Differences were declared significant at *P* ≤ 0.05. To investigate the dose effect of LEO and DEO on *E.coli* injected mice, the data were analyzed using the MIXED procedure of SAS 9.4 with model including EOs (LEO and DEO), doses of EO (0, 300, 600 and 1200 mg/kg of BW), and their interaction as fixed effects, and the random effect of animal. Contrasts were generated to compare the verage of the four EOs doses. The effect of increasing EOs dose was examined through linear and quadratic orthogonal contrasts using the CONTRAST statement of SAS. Differences were declared significant at *P* ≤ 0.05. The graphs of antioxidant indexes, inflammatory factors and tight junction proteins were drawn by GraphPad prism 7.0 software.

## Conclusions

The pretreatment of LEO and DEO had a protective effect on intestinal barriers function of *E. coli* infected mice. Both LEO and DEO had beneficial effects on preventing intestinal injury and inflammatory response by reducing the production of proinflammatory factors, increasing the concentration of antioxidant indexes and maintaining the integrity of duodenum. Whereas the LEO appeared to have higher anti-inflammatory activity than that of DEO. Our results suggest that as the main active componebt of LEO, the d-limonene not only played the major role in anti-inflammatory activities, but an increase the activity is expected in combination with other active components of LEO. Our work will shed light on developing new additive with anti-inflammatory activity from LEO.

## Data Availability Statement

The original contributions presented in the study are included in the article/supplementary materials, further inquiries can be directed to the corresponding author/s.

## Ethics Statement

The animal study was reviewed and approved by the Laboratory Animal Ethics Committee of Beijing University of Agriculture.

## Author Contributions

YLL cover the project. CZ designed and performed the experiments and analyzed the raw data. ZZ and DCN assisted with the experiments. All authors have read and agreed to the published version of the manuscript. All authors contributed to the article and approved the submitted version.

## Funding

This work was supported by Capital Science and Technology Innovation Voucher of Beijing, China (BNCXQ202004).

## Conflict of Interest

The authors declare that the research was conducted in the absence of any commercial or financial relationships that could be construed as a potential conflict of interest.

## Publisher's Note

All claims expressed in this article are solely those of the authors and do not necessarily represent those of their affiliated organizations, or those of the publisher, the editors and the reviewers. Any product that may be evaluated in this article, or claim that may be made by its manufacturer, is not guaranteed or endorsed by the publisher.

## Abbreviations

DEO: d-limonene essential oil
Eos: Essential oils
*E. coli*: *Escherichia coli*
IL-6: Interleukin-6
IL-1β: Interleukin-1β
LEO: Lemon essential oil
MDA: Malondialdehyde
MPO: Myeloperoxidase
RT-PCR: Real-time polymerase chain reaction
SOD: Superoxide dismutase
TNF-α: Tumor necrosis factor alpha
ZO: Zonula occludens.
